# Interaction between surface waves on wire lines

**DOI:** 10.1098/rspa.2020.0795

**Published:** 2021-02

**Authors:** Daniel Molnar, Tobias Schaich, Anas Al-Rawi, Mike Payne

**Affiliations:** ^1^ Theory of Condensed Matter Group, Cavendish Laboratory, University of Cambridge, Cambridge CB3 0HE, UK; ^2^ BT Labs, Adastral Park, Martlesham Heath, IP5 3RE Ipswich, UK

**Keywords:** electromagnetic surface wave, millimetre wave technology, computational electromagnetism

## Abstract

This paper investigates coupling between electromagnetic surface waves on parallel wires. Finite-element method (FEM)-based and analytic models are developed for single- and double-wire Sommerfeld and Goubau lines. Models are validated via measurements for Goubau lines and a comparison between the analytic and the FEM-based computations for coupled Sommerfeld- and Goubau-type lines is carried out. The measurements and calculations show remarkable agreement. The FEM-based and analytic models match remarkably well too. The results exhibit new favourable effects for surface waves propagation over multiple conductors. The short-range behaviour of the coupled wires and, consequently, the existence of an optimum separation of coupled wires is one of the most significant findings of this paper. We comment on the relevance of our results, particularly in relation to applications of high bandwidth demands and cross-coupling effects.

## 1. Introduction

Electromagnetic surface waves (SWs) of various kinds are attracting considerable attention owing to their potential for many applications from telecommunication to plasmonics [1–4]. Importantly, these emerging techno- logies have also been recently studied as backhaul solutions to the network standard 5G [5,6]. In this paper, we focus on SWs on wire conductors and particularly on the coupling effects between parallel single-wire conductors. Cylindrical SWs, as a solution to Maxwell’s equations, were first proposed by Sommerfeld over a century ago [7,8], and these solutions were reexamined most notably by Goubau and also by others from the 1950s onwards [9,10].

It was found that it is possible for a propagating SW mode to exist on a wire if the wire has finite conductivity (Sommerfeld case), or if corrugations are introduced on the surface of the wire or if the wire is coated by a dielectric. The last two modifications lower the effective conductivity of the conductor, making the mode confined to the surface of the wire [9]. The introduction of corrugations is known in the modern literature as ‘spoof plasmons’ [11] and applying a dielectric coating has become known as the Goubau or G line. Standalone or single-wire waveguides have been investigated since their discovery and their propagation characteristics have been studied in detail [9,12–15]. Recently, interest has been rekindled because of the possible application of these single-conductor lines in large bandwidth applications, owing to their near dispersionless and low loss characteristics [16]. Moreover, twisted copper wire pairs carrying SWs have also been proposed as possible alternatives to fibre networks for high-speed, multi-gigabit per second data transmission rates [17]. The interest in thus reusing telephone lines is motivated by the substantial cost benefits compared with replacing them by fibre optics. Without loss of generality in the following studies, we use the dimensions and material parameters specific for the UK telephone network [18]. However, our qualitative conclusions should equally apply to wires which have different dimensions from the ones considered in this work. In this article, we investigate the SW coupling effects and report our results for two parallel wires, for both the Sommerfeld (uncoated) and the Goubau (dielectric coated) wires. We first employ the so-called small-wire assumption (weak coupling) and then compare the results with finite-element method (FEM) numerical calculations (strong coupling). In the latter approach, no explicit assumptions are made about the wires’ separation or their diameters. We will concentrate on the frequency range of up to 300 GHz, which is relevant for the latest technologies and, most notably, will be in use for the 5G network [19].

## 2. Results

### (a) Single-wire waves

**Figure 1.**
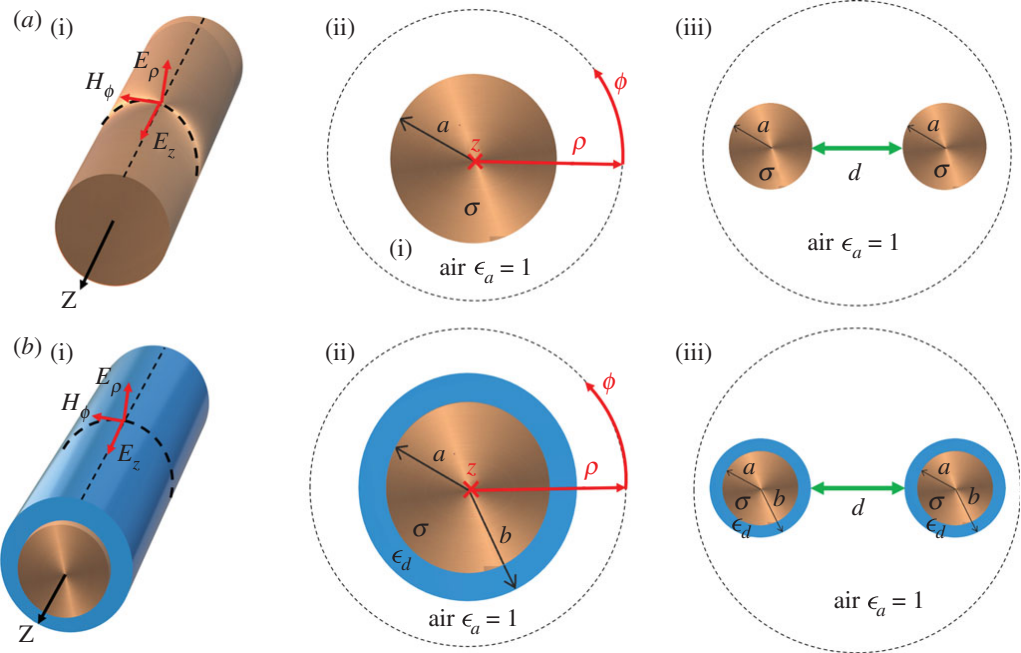
Schematic illustration of (*a*) the Sommerfeld wire (uncoated) and (*b*) the Goubau (coated) wire, with the field components. In (*a*(ii) and *b*(ii)) the coordinate system used in this paper is shown for both types with the cross sections of the wires. The wire radius *a* and the wire radius plus coating thickness *b* are indicated in the cross section. The material properties, the conductivity of copper, *σ*, the relative permittivity of air, *ϵ* and the relative permittivity of the dielectric coating of the wire, *d*, are also shown. (*a*(iii) and *b*(iii)) A schematic illustration of the coupled Sommerfeld and Goubau wire wave systems; the separation distance between the wire waveguides, *d*, is also shown. (Online version in colour.)

First we focus on single-wire waves and we use the analytically calculated solutions to validate our FEM results. We study wires with dimensions and material properties adopted from [18] throughout this work as follows: wire radius *a* = 0.25 mm, wire radius including coating *b* = 0.54 mm, conductivity *σ* = 5.57 × 10^7^ S m^−1^, relative permittivity *ϵ*_*d*_ = 2.54 and loss tangent tan*δ* = 1 × 10^−4^. A schematic illustration of the wire and the cylindrical coordinate system as well as the two-dimensional cross section used in the FEM study for both types of wires are shown in figure 1. The field components according to the cylindrical system ($H_{\phi }$, $E_{\rho }$ and *E*_*z*_) are shown on the schematics of the wires. The schematic illustration of the cross section of the coupled systems is also shown in figure 1.

#### (i) Sommerfeld single-wire wave

The matching of the electric and magnetic field components, *E*_*z*_ and H${\;}_{\phi }$, respectively, on the surface of the conductor (i.e. at *ρ* = *a*) yields the two conditions from which the characteristic equation of the uncoated wire wave or Sommerfeld wave can be found (see [7–9] for details). We use an iterative scheme from [20] to find the complex roots of equation (2.1) using Matlab. In the following *J*_0_, *Y*_0_ are the zeroth-order Bessel functions of the first and second kind, respectively, and $H_{0}^{\left(1,2\right)}$ are the zeroth-order Hankel functions of the first and second kind [21,22], $H_{0}^{\left(1,2\right)}(\gamma \rho )=J_{0}(\gamma \rho )\pm iY_{0}(\gamma \rho )$, where the + and – signs denote the first- and second-order Hankel function, respectively,

2.1 $$\frac{\gamma H_{0}^{\left(1\right)}(\gamma a)}{\epsilon_{a}H_{1}^{\left(1\right)}(\gamma a)}=\frac{\gamma_{c}J_{0}^{\left(1\right)}(\gamma_{c}a)}{\epsilon_{c}J_{1}^{\left(1\right)}(\gamma_{c}a)}.$$

Furthermore, the lateral complex wavenumber in air *γ* and in the conductor *γ*_*c*_ are $\gamma =\sqrt{k_{0}^{2}\epsilon_{a}-\beta^{2}}$, $\gamma_{c}=\sqrt{k_{0}^{2}\epsilon_{c}-\beta^{2}}$, where *β* is the longitudinal complex wavenumber and *k*_0_ is the free space wavenumber, and *ϵ*_*a*_ and *ϵ*_*c*_ are the relative permittivities of the surrounding air and conductor, respectively. Note that, in the following, we use interchangeably the words wavevector and wavenumber.

#### (ii) Goubau single-wire wave

In the case of the Goubau wave, the equations can be similarly derived from the continuity of the corresponding field components and therefore a characteristic equation can be found (equation (2.2)), as shown in [9,12], to obtain the solution without any losses (dielectric or metallic conductive) with Matlab as in [20],

2.2 $$\frac{h}{\epsilon_{d}}\frac{Z_{0}(hb)}{Z_{1}(hb)}=-\frac{\gamma }{\epsilon_{a}}\frac{K_{0}(\gamma b)}{K_{1}(\gamma b)},$$

where the function Z is obtained from the Bessel functions *J*_*j*_ and *Y*_*j*_, *Z*_*j*_ (*hρ*) = *J*_*j*_ (*hρ*) − (*J*_0_ (*ha*)/*Y*_0_ (*ha*))*Y*_*j*_ (*hρ*), *j* = 0, 1. The function *K*_*n*_ with *n* = 0, 1 is the imaginary argument Hankel function obtained from the Hankel function as $K_{n}(u)=(\pi /2)i^{n+1}H_{n}^{\left(1\right)}(iu)$ for *u* > 0 [21,22]. Further the lateral wavevector in air *γ* and in the dielectric *h* are $\gamma =\sqrt{\beta^{2}-k_{0}^{2}\epsilon_{a}}$ and $h=\sqrt{k_{0}^{2}\epsilon_{d}-\beta^{2}}$, where *ϵ*_*a*_ and *ϵ*_*d*_ are the relative permittivities of air and the dielectric coating, respectively.

Once the lossless solution is found for either the Goubau- or Sommerfeld-type wave it can be used to obtain the longitudinal attenuation along the line perturbatively [20],

2.3 $$\alpha =\frac{P_{\text{loss}}}{2P_{\text{TR}}},$$

where *P*_loss_ is the loss power (both dielectric and metallic) and *P*_TR_ is the power transmitted by the wave.

#### (iii) Comparison between FEM and analytically calculated results for single-wire waves

**Figure 2.**
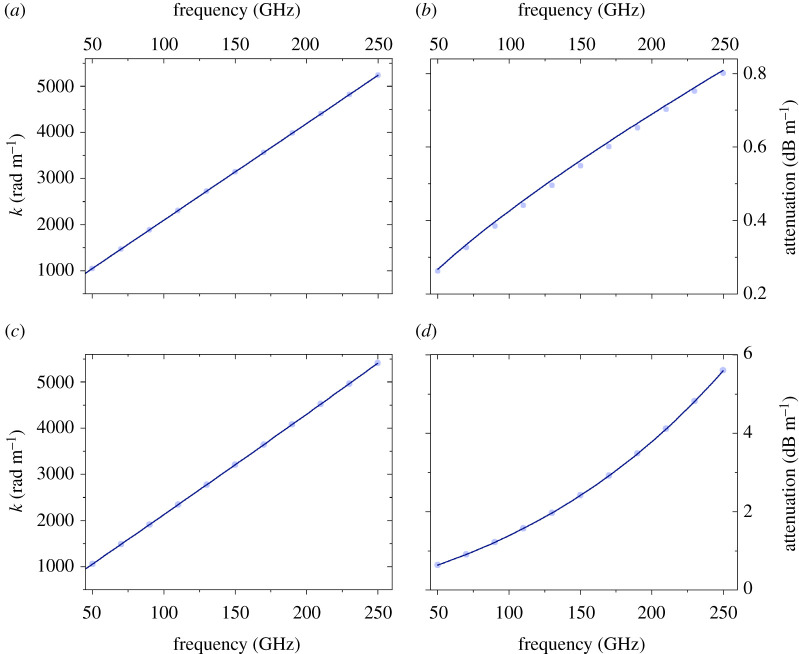
Comparison of the FEM computed (light blue dots) and analytically calculated (solid blue line). (*a*) The real part of the wavenumber (*k*) and (*b*) attenuation as a function of frequency for the Sommerfeld line. (*c*) The real part of the wavenumber (*k*) and (*d*) attenuation as a function of frequency for the Goubau line. Excellent agreement (less than 1% difference) is found between the results obtained by the two different methods.(Online version in colour.)

**Figure 3.**
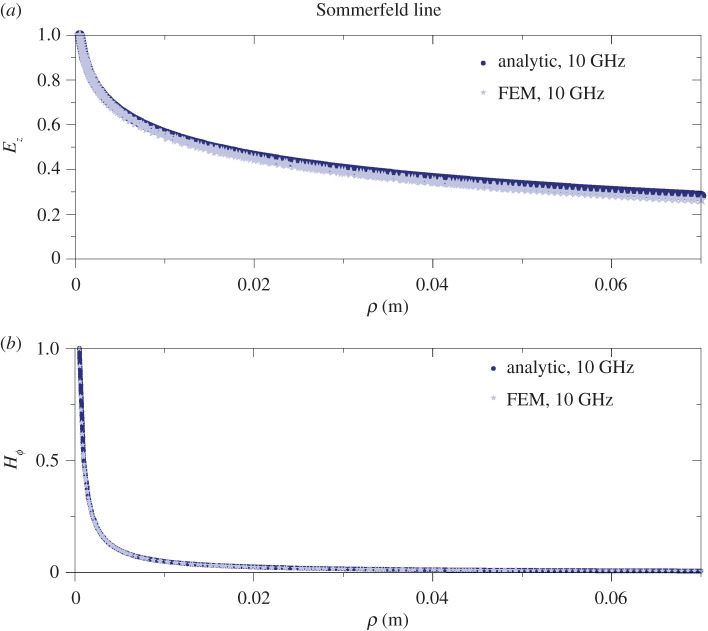
Comparison of the FEM computed (light blue stars) and analytically calculated (solid blue line) field components outside the wire (from *ρ* = *a*; see figure 1) for the Sommerfeld line at 10 GHz. (*a*) The *E*_*z*_ field component and (*b*) the $H_{\phi }$ field component as a function of the radial coordinate, *ρ*, in metres. Both *E*_*z*_ and $H_{\phi }$ are normalized to their value at the conductor surface.The two graphs are virtually indistinguishable. (Online version in colour.)

**Figure 4.**
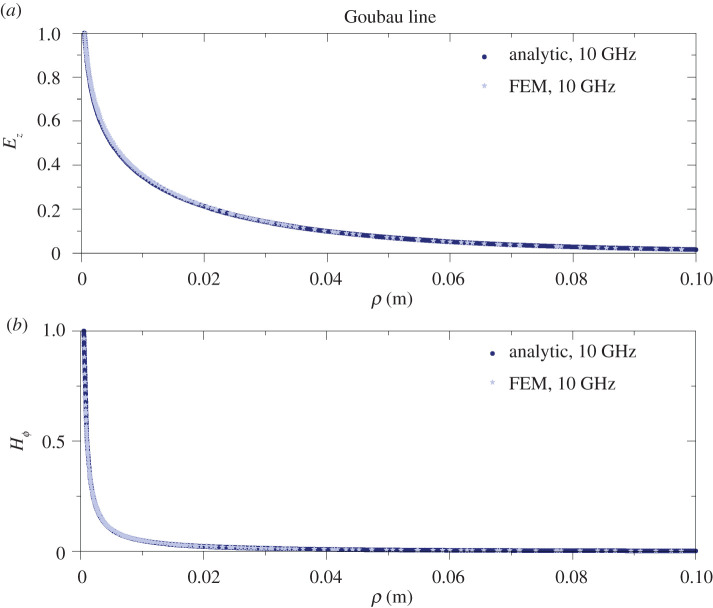
Comparison of the FEM computed (light blue stars) and analytically calculated (solid blue line) outside the wire (from *ρ* = *a*; see figure 1) for the Goubau line at 10 GHz. (*a*) The *E*_*z*_ field component and (*b*) the $H_{\phi }$ field component as a function of the radial coordinate, *ρ*, in metres. Both *E*_*z*_ and $H_{\phi }$ are normalized to their value at the conductor surface. The two graphs are again almost perfectly identical.(Online version in colour.)

We have solved the frequency domain Helmholtz (see equation (6) in appendix B) equation with the appropriate boundary conditions, using the commercial software COMSOL Multiphysics, to obtain the propagation characteristics of the SW (for more details, see appendix B). We compare our results obtained with the FEM solver with the results obtained from the semi-analytic (or, from now on for brevity, analytic approach) for the single-wire case. We focus on the dominant *E*_00_ mode only. We calculate with COMSOL the complex longitudinal wavenumber *β*, and from it the longitudinal attenuation *α*, i.e. the imaginary part of it, and the real wavenumber *k*, i.e. the real part of it, as a function of frequency in the range of 1–250 GHz. These quantities can be compared with the analytically obtained results. The results are plotted for both the Sommerfeld wire wave (uncoated wire) and the Goubau wire wave (coated wire) in figure 2. Excellent agreement is found between the results from the FEM numerical models and the analytically obtained results. The relative difference between the results obtained by the analytic and fully numerical methods is smaller than 0.5% in each case in the frequency range studied. Additionally, we also compare the field profiles, particularly the out-of-plane electric field *E*_*z*_ and the magnetic field $H_{\phi }$ as a function of the radial coordinate (*ρ*) for both types of wire waves. In both cases the amplitudes are normalized to the value of *E*_*z*_ and $H_{\phi }$ at the copper wire surface (*ρ* = *a* in figure 1). The relative difference between the results obtained with the FEM numerical calculation and the analytic results is within less than 0.5% in both cases. These plots are shown in figure 3 for the Sommerfeld-type wire and figure 4 for the Goubau-type wire.

### (b) Coupled surface waves

The analysis of the single lines can be extended to two axially parallel lines carrying an SW. Meyerhoff [23] first investigated the coupling of two weakly coupled SW lines, and then later Cook & Chu [24] carried out theoretical work in this topic. The latter article’s main interest was to extend Meyerhoff’s approach and particularly to consider higher-order modes and hybrid modes. In their paper, Cook and Chu only reported the lowest order mode TM_00_ coupling for two Goubau lines. More recently, Xu & Bosisio [25] carried out numerical calculations for two coupled SW lines and focused on the characteristic impedance of the lines as well as reporting propagation constants. They found that the even modes have a lower effective refractive index than the odd modes [25]. Furthermore, Xu and Bosisio only considered a fixed distance between the wires, and, therefore, fixed coupling strength. All of these works only considered the Goubau (coated wire) line. Little has been published on the coupling characteristics of Somerfeld-type lines. Additionally, Meyerhoff focused on the coupling between a single wire carrying an SW and a passive wire in the vicinity of it. Meyerhoff did not investigate the scenario in which both wires carry an SW, the two-wire wave case. In this work we investigate some of the propagation characteristics of the two-wire wave and map their dependence on the relative distance between the wires. We focus on the lowest order symmetric (even) and antisymmetric (odd) modes of the dominant *E*_00_ (or TM_00_) mode on each wire, and therefore the resultant two-wire wave. We investigate the coupling between two wires that have their *z*-axes parallel and are at distance *d* apart from one another. The geometry of the configuration is shown in figure 1. Note that the wires are identical and have the same dimensions and material properties as those used in the preceding sections in the case of single-wire waves.

#### (i) Analytic approach

In this section, we present the characteristic equation to find the complex propagation constant of the coupled wire wave. Note that for obvious reasons we will refer to this approach as analytic but in reality in the last step we performed numerical computation of the coupled characteristic equation, similar to the case of the single wire (see equations (2.1) and (2.2)). The most important assumption—namely that, outside the wires, the fields are simply the superpositions of the single wire waves—is due to Sommerfeld, who first suggested this type of coupling but did not present any results [8]. Furthermore, the currents in the wires are assumed to be azimuthally independent and the separation is assumed to be large between them compared with the wires’ radii. As we will see these latter assumptions will lead to the so-called weak coupling limit. We will focus on coupled identical wires in this work. Then, based on the above assumptions, the characteristic equation for the two-wire wave modes is found to be

2.4 $$\frac{\gamma }{\epsilon }\frac{H_{0}(\gamma a)\pm H_{0}(\gamma d)}{H_{1}(\gamma a)}=\frac{\gamma_{c}J_{0}(\gamma_{c}a)}{\epsilon_{c}J_{1}(\gamma_{c}a)},$$

where + is for the symmetric and − is for the antisymmetric two-wire mode. *a* and *d* are the wires’ radii and the distance between their centres, respectively (figure 1). *ϵ* and *ϵ*_*c*_ are the (complex) permittivity of the surrounding air and the conductor; *H*_0,1_ are the zeroth- and first-order Hankel functions, respectively; and *J*_0,1_ are the zeroth- and first-order Bessel functions, respectively. *γ* and *γ*_*c*_ are the lateral complex propagations constant in the surrounding medium (air) and the metal, respectively. For a detailed derivation of this characteristic equation, see appendix A.

This equation can be solved numerically using Matlab and therefore the complex wavenumber *β* of the coupled SWs can be obtained. This method can be used to study the propagation characteristics of two parallel Goubau lines as well. The procedure is somewhat more involved in that case, as first one assumes perfect metals and lossless dielectrics, and then in the second iteration the effects of these losses are taken into account. However, the assumptions of this analytic approach for the coupling in the limit of small separation distances between the wires result in neglecting contributions that are significant at small separations (see Discussion). Therefore, we expect the results to deviate from the ones obtained by FEM at small distances for both coated and uncoated wires.

#### (ii) FEM numerical results

**Figure 5.**
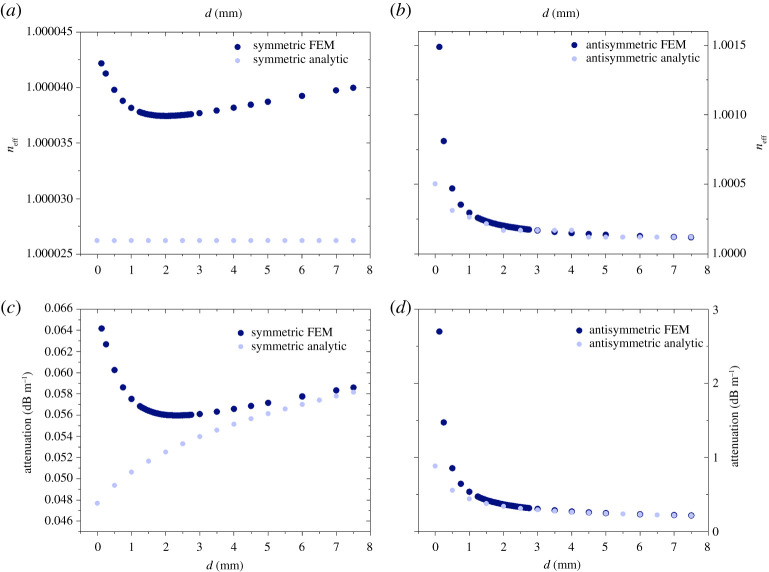
Comparison between the FEM (dark blue) and the analytically (light blue) computed results for the symmetric mode’s (*a*) effective index and (*c*) attenuation for two coupled Sommerfeld lines at 10 GHz as a function of separation of the wires. Comparison between FEM (dark blue) and the analytically (light blue) computed results for the antisymmetric mode’s (*b*) effective index and (*d*) attenuation for two coupled Sommerfeld lines at 10 GHz as a function of separation of the wires.(Online version in colour.)

**Figure 6.**
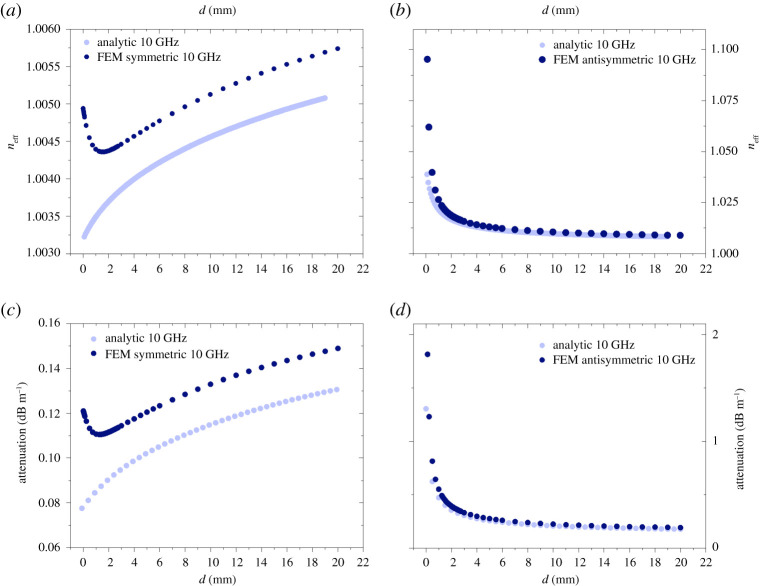
Goubau two-wire wave effective index computed by the FEM (dark blue) analytic (light blue) methods. (*a*) Symmetric mode and (*b*) asymmetric mode at 10 GHz as a function of separation between the two wires (*d*) in millimetres. Goubau two-wire wave attenuation calculated by the analytic and FEM methods for the (*c*) symmetric and (*d*) antisymmetric mode at 10 GHz as a function of separation betweenthe two wires (*d*) in millimetres. (Online version in colour.)

Following the analytic approach for the coupling presented in the previous section, we investigate the same configuration numerically, with the use of FEM. The boundary conditions are similar to the ones used in the single-wire wave cases, however now we also use the symmetry inherent in the geometry for the two-wire wave case [25]; for further details, see appendix B. We compare our results obtained using the analytic approach described previously with FEM computations. We expect the two results to asymptotically approach each other in the limit of large separations. We did not stipulate any special assumptions on the separation of the wires or about the tangential field in the FEM models. Therefore, we are particularly interested in the differences that may be present owing to the analytic assumptions. For large separations, it can be seen in figure 5 that the results obtained by the two methods are in very close agreement. However, for small separations the symmetric mode significantly differs between the results obtained analytically and those from FEM (compare figure 5*a* with figure 5*c*). In fact, the FEM calculation produces a characteristic shape of the attenuation curve with a clear, global minimum as a function of the separation of the wires. This global minimum is located at a specific separation between the wires, which we will call the optimum distance. We expect this optimum distance to be a function of the frequency of the wave because the fields’ radial extension is a function of frequency. We present the frequency dependence of this optimum distance later in the paper. The antisymmetric mode does not show such characteristics. In the antisymmetric case, the difference between the methods manifests itself in a larger predicted attenuation for small separations in FEM than for the analytic approach, but the overall shape is very similar with either method and the numerical values are very close (figure 5*b*,*d*). It can be appreciated immediately from the figure that the symmetric coupling case has much lower attenuation than the antisymmetric one. The difference is about 3 dB m^−1^ at the smallest separation distance and at the optimum separation distance it is 0.25 dB m^−1^, at which separation the symmetric mode’s attenuation is around 0.5 dB m^−1^. At very small separations in both cases, we observe a diverging attenuation, as the distance between the wires goes to zero. In addition, the antisymmetric mode’s attenuation approaches a certain value from above, whereas the symmetric mode’s attenuation approaches a certain value from below asymptotically for large separation distances. It is also clear that, apart from short separations, both the symmetric and antisymmetric modes’ attenuations asymptotically approach the values calculated analytically for the weak coupling limit. As a matter of fact, the deviation from it at small distances is the reason why we refer to the FEM calculated approach as the strong coupling limit. We leave a detailed explanation of these characteristics for the Discussion section of this paper. For the coupling of Gobau-type wires the resulting attenuation and effective mode indices at 10 GHz are plotted in figure 6. The overall shape of the curves is very similar to that of the two coupled Sommerfeld wires. Importantly, the global minimum of the attenuation constant as a function of frequency still exists; therefore, the optimum distance between the wires can be calculated for each frequency. For the Goubau wire wave, the fields’ radial extension at any given frequency is smaller than that of the Sommerfeld wire wave (with the same material and geometry parameters of the metal), hence the optimum distance is closer to 0 mm at 10 GHz than in the case of the uncoated wire. The difference between the results calculated by the analytic and FEM methods is larger in this case than for the Sommerfeld wire. It is especially obvious for the symmetric mode where the relative difference is as large as 28% in the attenuation, and this difference remains constant for large separations unlike in the case for the Sommerfeld two-wire waves. Additionally, the analytic method again misses important features of the close proximity effects at small separations for the symmetric mode. These will be elaborated in detail in the Discussion section of this paper.

#### (iii) Frequency dependence of the optimum distance between the wires

**Figure 7.**
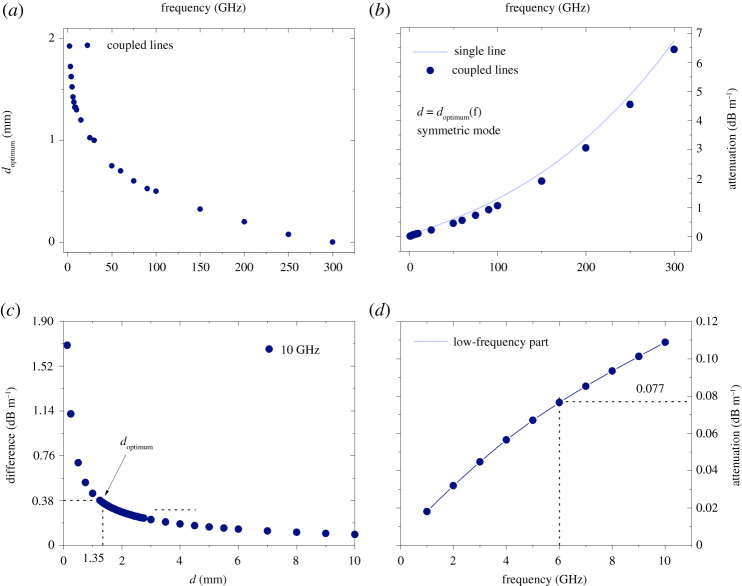
(*a*) Optimum separation (*d*_optimum_) of two coupled Goubau lines as a function of frequency for the symmetric mode. (*b*) Two Goubau lines’ symmetric mode attenuation at optimum separation compared with the attenuation for a single line as a function of frequency. (*c*) Difference in attenuation between the differential and symmetric modes of the Goubau two-wire wave at 10 GHz. The optimum separation for the symmetric mode at this frequency is 1.3 mm and the difference in attenuation between the modes for this separation is 0.38 dB m^−1^. (*d*) Two coupled Goubau lines at optimum separation distance as a function of frequency for the symmetric mode at low frequencies (up to 10 GHz). (Online version in colour.)

Importantly, it can be concluded from the symmetric mode attenuation curve that it is possible to find an optimum separation distance of the wires which minimizes the longitudinal propagation loss for both two coupled Sommerfeld and Goubau wire waves. It is expected that this distance would decrease as a function of frequency as the radial extension of the fields is smaller for higher frequencies for both types of waves [20]. The fact that such an optimum separation exists for the symmetric mode is probably one of the most important findings of this paper. In most real-life applications, the wires are coated and as such the fields’ radial extension is smaller than that of the Sommerfeld waves, as such the Goubau two-wave scenario is practically more relevant. Therefore, in this work, we only present the frequency dependence of the optimum distance in the symmetric mode for two coupled Goubau wire waves, up to 300 GHz. Importantly, it can be seen from figure 7*a* that for the Goubau line symmetric mode the optimum distance is 0 mm at 300 GHz. This holds significant practical importance since twisted pair telephone wires are in direct contact. However, it is known that SWs for single lines are sensitive to bends [26,27], and it is true that according to our knowledge the effect of twist has not yet been thoroughly investigated. Nevertheless, we believe our results support the proposal of using twisted pairs for SW transmission at these frequencies, because the diverging attenuation for the symmetric mode, as we describe in the Discussion, is partly inductive and that can be compensated by twisting. In figure 7*b*, the frequency dependence of the attenuation at the optimum distance for the symmetric Goubau mode is compared with that for a single-wire Goubau wave. The coupled waves offer an average gain of 0.4 dB m^−1^ in the 1–300 GHz frequency range. In figure 7*c*, the difference in attenuation between the symmetric and antisymmetric modes is plotted at 10 GHz as a function of separation between the wires. The optimum distance and the attenuation difference are also shown. Figure 7*d* is the low-frequency close-up of figure 7*b* and shows the practically important, unlicensed bands in Europe around 6 GHz.

#### (iv) Experimental results

**Figure 8.**
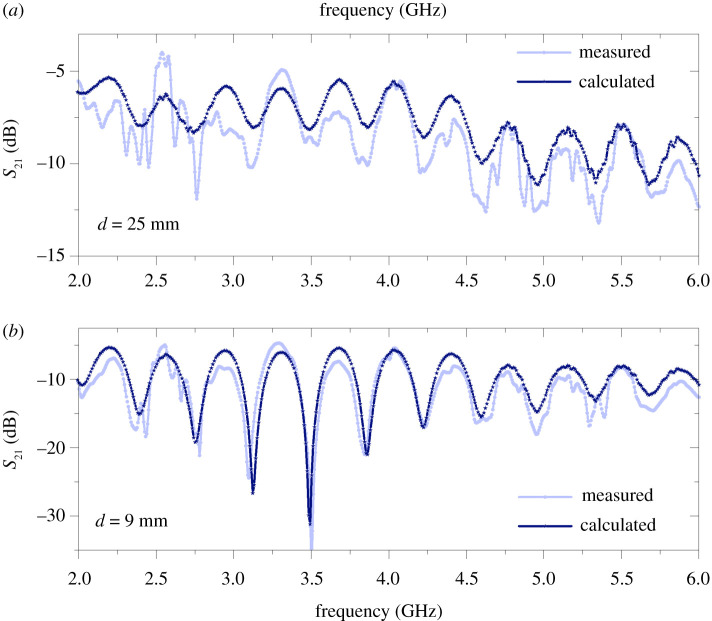
(*a*) Experimental (light blue line) and calculated (blue line and star) *S*_21_ parameters of coupled SWs on two Goubau wires for a separation of 25 mm. (*b*) Experimental (light blue line) and calculated (blue line and star) *S*_21_ parameters of coupled SWs on two Goubau wires for a separation of 9 mm.(Online version in colour.)

Measurements have been carried out on SWs on two parallel wires to experimentally validate the theoretical results. We have measured the system as a two-port device and have recorded its *S*-parameters (see appendix C). The wires are both coated (Goubau line) and have identical material parameters to the ones studied in the preceding sections, namely *a* = 0.25 mm and *b* = 0.54 mm (figure 1). Importantly, the thicker insulation and smaller metal cross section results in higher losses, thereby eliminating some practical issues with the noise floor. Nevertheless, we recalculated the attenuation for these parameters with the same methods as above, for all separations, and the comparison serves as validation of our theoretical results. We are comparing calculated and measured *S*_21_ parameters for different wire separations as a function of frequency in coupled SWs guided by coated (Goubau) wires. The measurement set-up is designed to measure the sum of symmetric and antisymmetric modes by using two extra coaxial cables on one side of each wire (see appendix C). Hence, the measured data alternate between the symmetric and antisymmetric eigenmode and are compared accordingly by taking the sum of the two modes in the theoretical model. The coaxial cables, however, cause the strong resonant behaviour observed in figure 8. Furthermore, to couple SWs onto the wires specially designed planar launchers were used together with a splitter circuit [28]. In the calculations we included the losses of the splitter circuits and launchers. Additionally, both the splitter circuits and the launchers have finite bandwidth over which their operation is optimized to maximize transmission (see appendix C). Taking this into account, we expect to find the closest agreement between calculation and measurement around 4 GHz. The results of the measurements and calculations are plotted for separations of 25 mm and 9 mm in figure 8*a*,*b*, respectively. It is clear that there is excellent agreement between calculation and measurement results for the 9 mm separation: the two curves follow the same qualitative shape and the difference is within a few dB across the whole frequency range. At 4 GHz, this corresponds to 1% relative difference between the measured and calculated values for the 9 mm separation. At 3.6 GHz and at 4.4 GHz, the relative differences are 17.5% and 20%, respectively. For the 25 mm separation between the wires the same qualitative statement is true; however, the noise is larger in this case even around 4 GHz. This stems from the larger decoupling of the two-wire wave. At 4 GHz, the relative difference is still very small, 1.05%. The agreement between theory and measurement is remarkable, within 1% at and around 4 GHz for both separations, where the launchers have their transmission peaks.

## 3. Discussion

The differences between the analytic approach and the FEM numerical calculations originate from the assumptions made in the former. Namely, at small separations the assumption that the field of the other wire at each wire’s metal surface and the tangential electric field are negligible. That is, each wire carries an SW independent of the other on the metal surface and inside the metal, and a simple superposition of the SWs outside of the wires is present.

However, in reality, the total current density at each wire’s surface can be written in the following form:

3.1 $$J_{\text{tot}}=J_{\text{SW}1}+J_{\text{ind}},$$

where ***J***_SW1_ is the self-current density due to the presence of a single SW on a wire and ***J***_ind_ is the induced current density by the other wire’s fields. The loss (conductor loss only for Sommerfeld lines) can be obtained by

3.2 $$P_{\text{loss}}=\oint \frac{1}{2}R_{s}|J_{\text{tot}}{|}^{2}\;\text{d}l.$$

**Figure 9.**
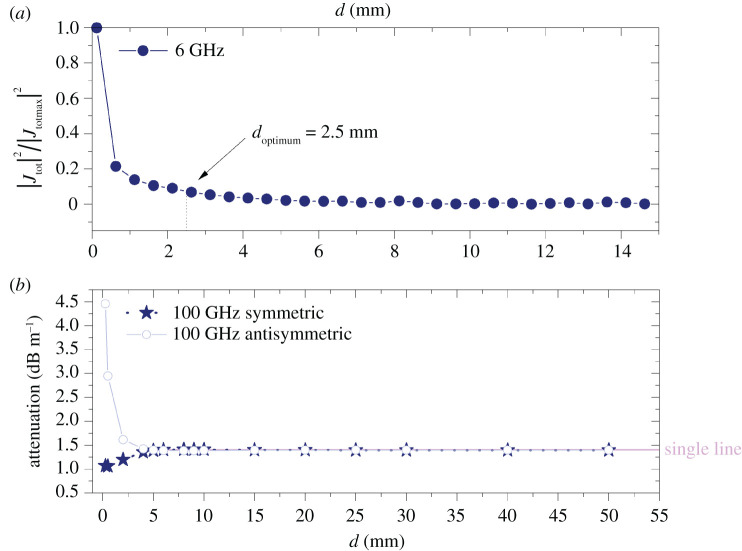
(*a*) The normalized total current density squared (which is proportional to *P*_loss_; see equation (3.2)) on the metal surface as a function of separation (*d*) between the wires in millimetres for two symmetrically coupled Sommerfeld lines. It can be seen that |*J*_tot_|^2^ is nearly constant for larger separations, which is expected as at large separations |*J*_ind_| is negligible and |*J*_SW1_| is constant throughout. Since in equation (3.1) only |*J*_ind_| depends on the relative distance between the wires, it has to cause the upturn in *P*_loss_. Ultimately, this leads to the total loss overcoming 2 × *P*_TR_ at sufficiently small separations, which causes the upturn in the attenuation observed for symmetrically coupled lines. The upturn is therefore only observed when *J*_ind_ is taken into account in the FEM models. (*b*) Large separation (*d*) behaviour of symmetric and antisymmetric modes at 100 GHz of two coupled Goubau lines. The horizontal line is the single-line equivalent attenuation at 100 GHz. Note that both the symmetric and asymmetric attenuation curves asymptotically converge to the single line’s attenuation. In the case of Goubau lines, the fields are more tightly confined than the Sommerfeld case at any given frequency and they converge to the single line at smaller separations. (Online version in colour.)

***J***_ind_ has two components which are driven by the fields ***E***_*T*_ and ***H***_*ϕ*_ (see figure 1 for the field components). In the analytic approach, i.e. in the first-order approximation, ***J***_ind_ is completely neglected and therefore the power loss in the metal is independent of the distance between the wires, since it only depends on one wire’s own current density. However, the $H_{\phi }$ component strongly depends on the distance between the wires as it is an approximately exponential function (from the large argument asymptotic form of the Hankel function) of the radial coordinate, as we have seen for the single wire (also see figures 3 and 4). As for the tangential electric field component ***E***_*T*_, its effect is again stronger at smaller distances, and it results in inducing a current density proportional to ***E***_*T*_, denoted as ***J***_*T*_, with opposing directions on the two halves of the wire. At large distances, these tend to cancel out and the net field is close to 0, thus its effect can be neglected (figure 9*a*). At small distances, this imbalance is greater and it has a non-zero contribution to the induced current. *P*_loss_ can be obtained by numerical integration in COMSOL; thus, one can see how it depends on the relative distance for a given frequency. Since the geometry is simply the circular cross section of a wire, the dependence of |***J***_tot_|^2^ on the separation between the wires is the same as that of *P*_loss_. |***J***_tot_|^2^ normalized to its maximum value at 6 GHz as a function of distance between the wires is plotted in figure 9*a*. Note that the vertical line is the optimum separation distance between the wires, where the upturn is observed in the attenuation coefficient. This is a clear indication that the upturn is caused by ***J***_ind_. Since the attenuation coefficient behaves similarly for all frequencies we expect this to be a qualitatively representative behaviour for all frequencies. In our analytic approximation (based on Sommerfeld [8]), the transmitted power by the wave (*P*_TR_) through a closed surface S can be obtained by the following surface integral:

3.3 $$P_{\text{TR}}=\int_{S}(E_{1}\pm E_{2})\times (H_{1}\pm H_{2})\;\text{d}A.$$

The plus sign is for the symmetric mode whereas the minus is for the antisymmetric mode. The cross products between *E*_*i*_ and *H*_*i*_ are the single wire’s Poynting vector, whereas the *E*_*j*_ and *H*_*i*_ (∀i&j∈1,2) are the two-wire wave Poynting vector. The two-wire terms are again dependent on the separation of the wires: the closer the wires the greater the coupling strength. Note that this expansion of the fields is only true in the weak coupling limit, where the two-SW is a superposition of single SWs. The attenuation is obtained similarly to the single-wire case (see equation (2.3)). Therefore, the attenuation will depend on the relative magnitudes of the two opposing effects: on one hand, a smaller distance will result in greater induced currents in the other wire; on the other hand, this will increase the power in the two-wire wave. It is clear from this why the weak coupling analytic approach does not produce an upturn in the attenuation at small separations seen in the case of symmetric coupling. It simply disregards the induced current parts at small distances and therefore *P*_TR_ in equation (2.3) gets larger and larger as the separation is reduced, whereas *P*_loss_ has no dependence on the relative distance between the wires in this approximation. The above reasoning also holds true for the Goubau two-wire wave, although the losses consist of dielectric as well as metallic losses. But essentially the same mechanism as detailed above can explain the behaviour of the attenuation curve as a function of separation between the wires. Additionally, there is a small constant difference (less than 10%) between the analytically and FEM obtained attenuation and the effective mode index in the symmetric mode throughout the separation range. This comes about through the dielectric loss calculation and the method employed in which the transmitted power in the dielectric is calculated from the single-wire case; therefore, it underestimates the dielectric loss slightly, so is best suited for thin but not too thin dielectrics (see appendix A). The electric and magnetic fields have smaller radial extent at any given frequency, resulting in a smaller optimum distance between the coated wires than between the two Sommerfeld wires. This results in a sharper decrease in the optimum distance as a function of frequency, and in the frequency range studied in this work the optimum distance between the coated wires already reaches 0 mm. It is worth mentioning that, although Cook and Chu reported two coupled Goubau lines’ effective mode indices as a function of separation for the lowest mode [24], their work can also be considered as a weak coupling limit for this specific case. This is evident from the fact that their method did not produce an optimum separation distance for the symmetric mode. The existence of an optimum distance for the symmetric mode is the result of strong coupling and we clearly obtain it with our numerical calculations.

**Figure 10.**
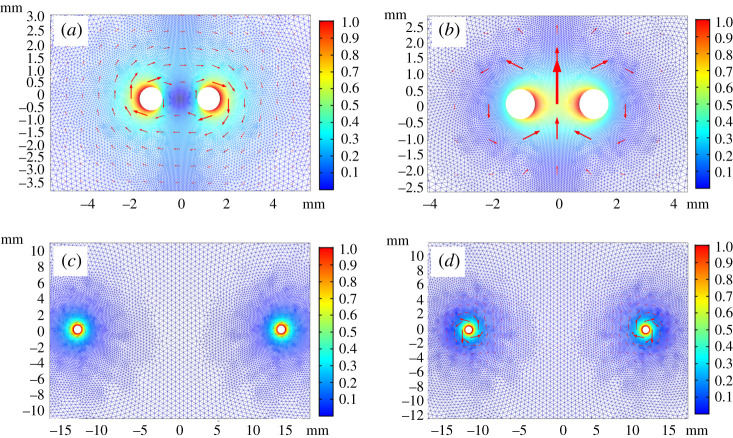
(*a*–*d*) The colour scale is normalized intensity of the total magnetic field to its value at the metal surfaces. Arrows depict the total (normalized) magnetic field with the length proportional to the magnetic field intensity. (*a*) Symmetric mode at a small separation distance (*d* = 1.5 mm) between the wires at 10 GHz in the Sommerfeld case. Note that only the outer part of the wires contributes to ‘binding’ the-wave in the two-wave mode when they are close. The key factor is the relation between two effects: the formation of the two-wire wave and the extinction of the fields on the wires’ inner sides. The latter effect results in increasing the losses as the separation is reduced and at smaller separations than the optimum distance dominates the attenuation. (*b*) Antisymmetric mode at small a separation distance (*d* = 1.5 mm) for two Sommerfeld wires at 10 GHz. Note that at this separation the field is concentrated between the wires; therefore, the effective surface area of the wire is greatly reduced compared with a single-wire case. In the antisymmetric mode, even in close range the two-conductor SW does not manifest; rather, the field profile resembles two single-wire SWs but with smaller cross section because of the reduced intensity of the field on the outer perimeter of the wires. (*c*) Symmetric mode at large separation distance (*d* = 30 mm) between the wires at 10 GHz for two Sommerfeld wires. At large separation distances between the wires the field profile looks as if two independent one-wire waves on each wire are propagated out of plane in the *z*-direction. (*d*) Antisymmetric mode at large separation distance (*d* = 30 mm) between the wires at 10 GHz for two Sommerfeld wires. At large separation distances between the wires the field profile looks as if two independent one-wire waves on each wire are propagated out of plane in the *z*-direction. Also note, however, that this is only true exactly in the infinite separation limit and there is a slight asymmetry still present in the field profile on the wire cross section at finite distances. (Online version in colour.)

The shape of the attenuation curves described above can also be understood from an intuitive picture. The attenuation will first decrease as *d* is decreased from large values, because a two-wire wave forms, which can be thought of as an SW with of the order of twice the effective cross section of a single wave. This is most obvious from the magnetic field’s arrow plot in figure 10*a*. However, as the wires get even closer in the symmetric mode the magnetic fields have opposite directions (in the *ϕ* direction) and this results in a magnetic field at the wire surfaces that induces an electric field. This, therefore, results in an induced current density that is in the opposite direction to the wire’s self-current density, thereby extinguishing the fields on the inner sides of the wires (figure 10*a*, intensity plot). This effectively reduces the metal cross section to which the field is ‘bound’ or increases the effective resistivity, hence increasing the losses (if the amplitude of current density of each wire is kept constant, which is the case here). In the analytic description, however, we neglect the effect of the fields caused by the other wire, at each of the wires’ metal surfaces. For large separation distances the fields resemble two non-interacting SWs on each wire (figure 10*c*,*d*, arrow plots) in both the antisymmetric and symmetric cases; therefore, the waves become ‘uncoupled’ at large separations. This is due to the fact that both electric and magnetic fields are negligible at large distances from the wires, as the field amplitudes decay approximately exponentially outside the wires [20,21] (also see figure 3 for the single wires’ field profile as a function of the radial coordinate). So, in equation (3.3), only the *E*_*i*_ and *H*_*i*_ components are non-negligible, or the single-wire wave contributions. For the antisymmetric mode, we expect the attenuation to increase as the separation of the wires is decreased, for both the analytic and the FEM numerical models. In this mode, the two-wire SW ‘cannot form’ even for small separations because of the opposing (*ϕ*) directions of the magnetic fields on each conductor (figure 10*b*,*d*, arrow plot). The field profile will always resemble that of two separate SWs at each wire, which can also be seen from equation (3.3). Therefore, as we gradually decrease the separation between the wires the attenuation gets larger because no two-wire wave forms, due to the opposite sign of the amplitude of the current density; in other words, the resulting two-wire fields are the difference of the fields produced by each wire. This is true in both the analytic and FEM models. In the analytic approach, the fields and the induced current caused by the other wire at each of the wires’ surfaces at small separations are neglected, but in reality the fields strongly interact and couple in a manner such that the effective resistance of the wires increases. In figure 10*b*, a small separation distance is shown. It can be seen that the field is concentrated between the two wires; therefore, the field only ‘binds’ to a fraction of the inner sides of the wires. Hence, we expect the analytic model to underestimate the losses at small separations, which we can see, for instance, in figure 5*b*. At large distances, the field profile will again resemble two non-interacting SWs on each wire, as can be seen in figure 10*d*. Therefore, the attenuation will asymptotically approach the attenuation of the single-wire SW from above. As we have shown, for large separation distances, both modes’ attenuations ought to approach the attenuation of the single-wire SW at any given frequency. As expected, the antisymmetric mode will asymptotically approach it from above and the symmetric mode from below as a function of the separation distance. At 100 GHz, this can be clearly seen from the attenuation plot in figure 9*b*. The intuitive picture presented above also applies to the coupled Goubau wire waves; however, there are slight modifications. It is instructive to think of the Gobau line as a Sommerfeld line surrounded by a medium other than air. Since the wires are coated the metal surfaces cannot get arbitrarily close to each other, which would result in very large losses as described above for the Sommerfeld wire. Then at very high frequencies the single-wire wave’s propagation constant will approach the propagation constant of the dielectric as the SW becomes very closely bound to the dielectric [9]. Therefore, at sufficiently high frequencies the optimum separation for Goubau wires has to be zero, because the metal surfaces can never get too close to cause the upturn in the attenuation that we described above for the Sommerfeld wires. This argument holds for not too thin (in terms of the wavelength and relative to the wire’s diameter) dielectric coatings, which are the ones we consider in this work. From our calculations, we also conclude that at 300 GHz the optimum separation of the coated wires is 0 mm.

## 4. Conclusion

In this paper, we have developed FEM-based and analytic models to uncover unexplored properties of coupled SW lines. We considered both Sommerfeld and Goubau lines to investigate coupling in dual uncoated and coated lines, respectively. Both models have been validated against each other in both the single- and two-line scenarios, providing remarkable agreement within 0.5% for Sommerfeld wires except for the case of symmetrically coupled wires at very small separations (less than 2 mm or 4 wire radii). For Goubau wires, the differences are slightly larger in the symmetric coupled case; however, for the antisymmetric and single-wire cases the differences are similarly small. Our results suggest that antisymmetric SWs can suffer attenuation losses as high as 3 dB m^−1^ while a symmetrically coupled SWs exhibit significantly lower attenuation of 0.064 dB m^−1^ in the Sommerfeld case at 0 mm separation at 10 GHz, which is a more than 40% reduction in attenuation. The effects of coupling in the Goubau case are less dramatic than those in the Sommerfeld case with attenuations as high as 2 dB m^−1^ in the antisymmetric mode and 0.11 dB m^−1^ in the symmetric mode at 1 mm separation, i.e. a 25% reduction in loss. This is due to the fact that the fields are more confined laterally and do not expand as far as in the Sommerfeld scenario. The results, via both FEM-based and analytic models, additionally demonstrated the convergence of the attenuation loss to the single-wire case when coupling occurred over large separation distances. We have also validated our analytic results by means of experiments on coupled SWs. The measured and calculated results show remarkable agreement at 4 GHz within 1% relative difference of each other. The findings of this paper identify the nature and the role of coupling in determining the losses of the SW mode in multi-conductor environments particularly for both short- and long-range communication systems. These results are novel and the coupling of SWs on Sommerfeld lines has not previously been investigated thoroughly to the authors’ knowledge; in particular, the frequency dependence of either coated or uncoated wires has not been mapped. The short-range effects, especially the upturn of the attenuation for symmetric waves, uncovered by the numerical results is an entirely novel finding of this paper for coupled SWs on both Sommerfeld and Goubau wires. This short-range effect can prove crucial in large bandwidth applications of SWs and is the most important finding of this paper as well as a novel effect for SWs.

## Figures and Tables

**Figure 11.**
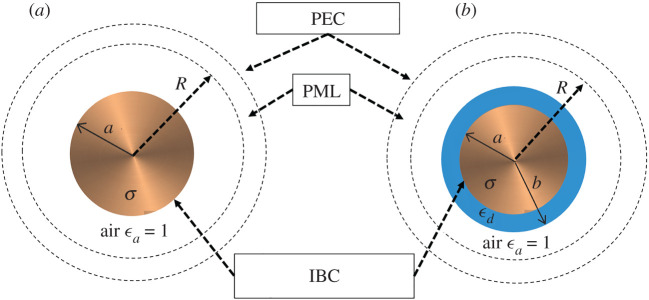
Cross-section schematics to show the boundary and domain conditions used in the FEM models of single wires in this work. (*a*) Sommerfeld wire and (*b*) Goubau wire. (Online version in colour.)

**Figure 12.**
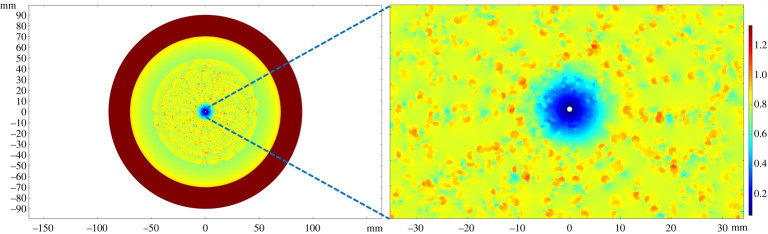
Mesh used in this work for a single Sommerfeld-type line at 10 GHz. The colour bar indicates the mesh size in millimetres, whereas the elements are coloured according to the largest side of the element. (Online version in colour.)

**Figure 13.**
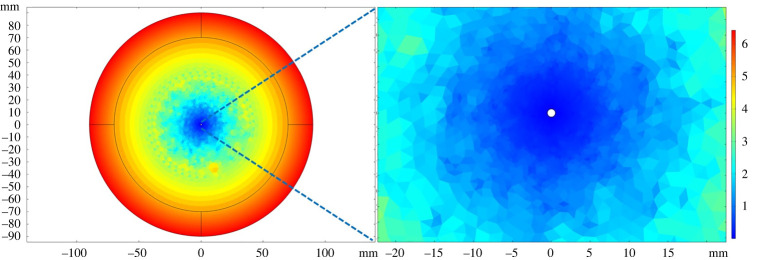
Mesh used in this work for a single Goubau-type line at 10 GHz. The colour bar indicates the mesh size in millimetres, whereas the elements are coloured according to the largest side of the element. (Online version in colour.)

**Figure 14.**
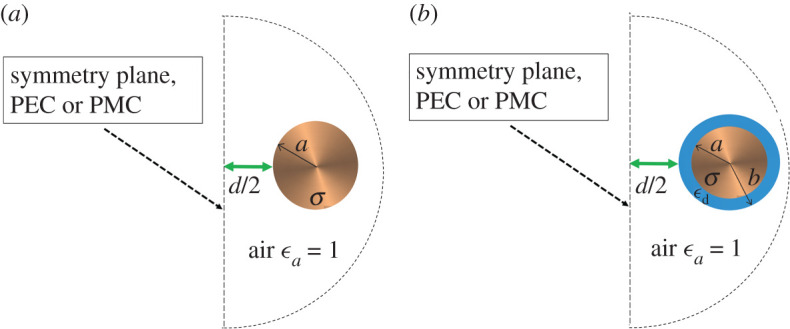
Cross-sectional schematics to show the symmetry boundary used in the FEM models for coupled wire waves in this work. The dashed lines represent the computational domains. Other details of the boundary and domain conditions, such as the PMLs, are not shown, but are identical to the single wire shown in figure 11. (*a*) Sommerfeld wire and (*b*) Goubau wire. (Online version in colour.)

**Figure 15.**
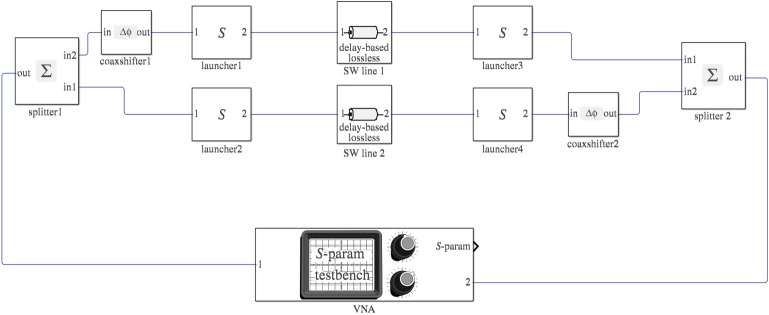
Schematic diagram of the measurement set-up. The launchers, shifters and splitters were also measured separately to make sure that their effect can be subtracted from the entire system’s *S*-parameters. In this way, we could compare our theoretical results with experiments for the SW.

**Figure 16.**
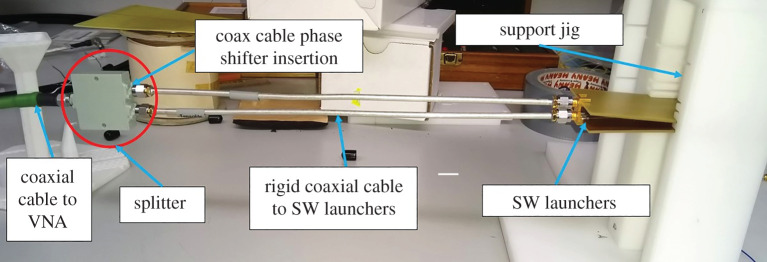
Picture of the measurement of the *S*-parameters of coupled SWs. Although the ‘phase shifting’ extra coaxial cable is not shown the place of insertion is indicated. (Online version in colour.)

**Figure 17.**
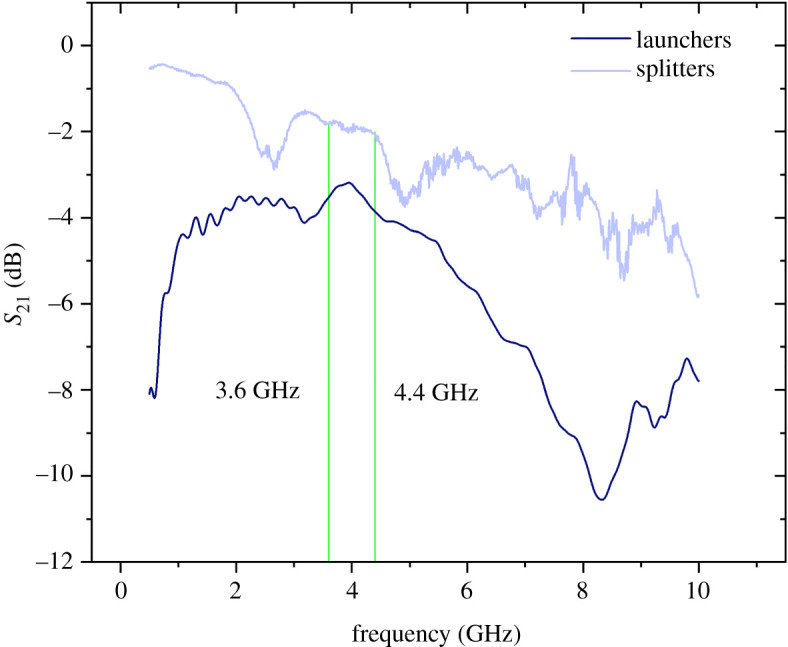
*S*_21_ measurement results for a pair of launchers and a pair of splitters. The latter include the losses of the four connecting rigid coaxial cables. The vertical lines indicate the range in which the launchers have peak transmission and the splitters have close to linear behaviour as a function of frequency. In this region, we can expect that simply subtracting these *S*_21_ parameters from the *S*_21_ data for the whole set-up (figure 15) will result in the two-wire wave’s *S*_21_ alone. (Online version in colour.)

## Data accessibility

The experimental data, analytic simulation data and source code may be obtained from [29].

## Appendix A. Derivation of the characteristic equation from first principles, analytic approach

In the following, we derive the characteristic equation to find the complex longitudinal wavenumber (*β*) of the coupled wire wave. Note that we will refer to this approach as analytic but in reality in the last step we make use of a numerical solution of the coupled characteristic equation, just as we did when we solved for the single wire.

We use two cylindrical coordinate systems (*r*; *ϕ*; *z*) and (*r*^′^; *ϕ*^′^; *z*^′^) with their respective centres in the middle of the conductors and their *z*-axes parallel. The fields are assumed to be a superposition of the single-wire SW outside and a standard single-wire SW inside the conductors. The conductors are assumed to be made of the same material and both have radius *a*. A common factor of e^i(*ωt*−*βz*)^ is suppressed in this derivation. We split the electric field into a transverse field ***E***_*T*_ and a longitudinal field ***E***_*z*_ such that the total field ***E*** is given as $\vec{E}=\vec{E_{T}}+E_{z}\hat{z}$, where $\hat{z}$ is the unit vector in the *z*-direction. For the magnetic field ***H***, we can express the fields outside the conductors as follows:

A 1 $$\begin{array}{rl} & E_{z}=B_{1}H_{0}(\gamma r)e_{z}+B_{2}H_{0}(\gamma r^{\prime })e_{z^{\prime }},\\ & H_{\phi }=\frac{\text{i}\omega \epsilon }{\gamma }(B_{1}H_{1}(\gamma r)e_{\phi }+B_{2}H_{1}(\gamma r^{\prime })e_{\phi^{\prime }}),\\ & E_{T}=\frac{\text{i}\omega \beta }{\gamma }(B_{1}H_{1}(\gamma r)e_{r}+B_{2}H_{1}(\gamma r^{\prime })e_{r^{\prime }})\\ \;\text{and}\; & \gamma =\sqrt{\omega^{2}\epsilon \mu -\beta^{2}}, \end{array}\}$$

where *ϵ* and *μ* are the permittivity and permeability of the surrounding medium and *B*_1,2_ are arbitrary constants to be determined. *H*_0,1_ are the zeroth- and first-order Hankel functions, respectively, and *γ* is the lateral complex wavenumber in the surrounding medium. Inside the conductors we have the following relations:

A 2 $$\begin{array}{rl} & E_{z}=A_{1}J_{0}(\gamma_{c}r)e_{z}+A_{2}J_{0}(\gamma_{c}r^{\prime })e_{z^{\prime }},\\ & H_{\phi }=A_{1}\frac{\text{i}\omega \epsilon_{c}}{\gamma_{c}}J_{1}(\gamma_{c}r)e_{\phi }+A_{2}\frac{\text{i}\omega \epsilon_{c}}{\gamma_{c}}J_{1}(\gamma_{c}r^{\prime })e_{\phi^{\prime }},\\ & E_{T}=A_{1}\frac{\text{i}\beta }{\gamma_{c}}J_{1}(\gamma_{c}r)e_{r}+A_{2}\frac{\text{i}\beta }{\gamma_{c}}J_{1}(\gamma_{c}r^{\prime })e_{r^{\prime }}\\ \;\text{and}\; & \gamma_{c}=\sqrt{\omega^{2}\epsilon_{c}\mu_{c}-\beta^{2}}, \end{array}\}$$

where *ϵ*_*c*_ and *μ*_*c*_ are the permittivity and permeability of the conductor, respectively. *J*_0_ and *J*_1_ are zeroth- and first-order Bessel functions, respectively, and *γ*_*c*_ is the lateral complex wavenumber in the metal. Let *d* be the distance between the centres of the two conductors and hence the distance between the centres of our two coordinate systems, that is, the unprimed and primed ones. We assume that the conductors are small such that the fields originating from one conductor do not vary significantly over the other. In addition, if *d* ≫ *a*, where *a* is the radius of the conductors, these fields will be small and can be neglected. Hence, we can formulate approximate boundary conditions by only considering the perturbing effect of the other conductor on the longitudinal electric field. These assumptions constitute what we call the weak coupling approximation. This was first suggested by Sommerfeld for the SW problem [8], but he never published a solution. These assumptions result in the following equations:

A 3 $$\begin{array}{rl} & B_{1}H_{0}(\gamma a)+B_{2}H_{0}(\gamma d)=A_{1}J_{0}(\gamma_{c}a)\\ \;\text{and}\; & B_{1}H_{0}(\gamma d)+B_{2}H_{0}(\gamma a)=A_{2}J_{0}(\gamma_{c}a) \end{array}\}$$

and

A 4 $$\begin{array}{rl} & B_{1}\frac{\text{i}\omega \epsilon }{\gamma }H_{1}(\gamma a)=A_{1}\frac{\text{i}\omega \epsilon_{c}}{\gamma_{c}}J_{1}(\gamma_{c}a)\\ \;\text{and}\; & B_{2}\frac{\text{i}\omega \epsilon }{\gamma }H_{1}(\gamma a)=A_{2}\frac{\text{i}\omega \epsilon_{c}}{\gamma_{c}}J_{1}(\gamma_{c}a). \end{array}\}$$

Note that we only take into account the longitudinal electric field, which is a first-order approximation. To solve these equations, first we find the condition $B_{1}^{2}=B_{2}^{2}$. Without loss of generality, we may choose *B*_1_ = 1. Then we have the two options *B*_2_ = 1 or *B*_2_ = −1 corresponding to a symmetric and an antisymmetric mode, respectively. It is easy to show that for the symmetric case *A*_1_ = *A*_2_ and for the antisymmetric case *A*_1_ = −*A*_2_. Finally, the characteristic equation for the two modes is then found to be

A 5 $$\frac{\gamma }{\epsilon }\frac{H_{0}(\gamma a)\pm H_{0}(\gamma d)}{H_{1}(\gamma a)}=\frac{\gamma_{c}J_{0}(\gamma_{c}a)}{\epsilon_{c}J_{1}(\gamma_{c}a)}.$$

The positive sign corresponds to the symmetric, the negative sign to the antisymmetric mode. As in the case for the single Sommerfeld wire, we can make use of the large argument expansion for the Bessel function to find (*J*_0_ (*γ*_*c*_ *a*))/(*J*_1_ (*γ*_*c*_ *a*)) ≈ *i*. The transcendental equation is then solved with standard numerical methods by using Matlab, and hence the lateral complex wavenumber (*γ*) and, from it, the longitudinal complex wavenumber (*β*) can be obtained. For the two Goubau lines, the procedure is slightly modified. First, a lossless dielectric and a perfect metal are assumed and then the losses are added. Finally, the longitudinal attenuation is calculated from *α* = *P*_loss_/2 *P*_TR_, identical to the single-wire case; the details for this calculation can be found, for example, in [20]. Note that our assumptions limit the accuracy of the calculations to thin dielectric coatings at present. The assumptions are valid as long as the conductor does not enter the plasmonic region, or more precisely when in equation (6) *σ*/*ω* becomes comparable to *ϵ*_0_. If we assume that the transition from metallic behaviour to plasmonic happens at 3 THz, the thickness of the coating has to be >10 *μ*m.

## Appendix B. FEM numerical model

To model SW propagation on a single wire and, later, on coupled wires we use the commercial FEM code COMSOL Multiphysics. The frequency domain Helmholtz equation is solved (see equation (6)), where the ${\text{e}}^{\text{i}(\omega t-\beta \hat{z})}$ dependence is assumed throughout but suppressed in notation only. By solving this equation, the out-of-plane component of the electric field is obtained and the rest of the field components are obtained from Maxwell’s equations,

B 1 $$\nabla \times \mu_{r}^{-1}(\nabla \times E)-k_{0}^{2}\left(\epsilon_{r}-\frac{\text{i}\sigma }{\epsilon_{0}\omega }\right)E=0,$$

where *k*_0_ is the free space wavenumber for a given angular frequency *ω*, *σ* is the electrical conductivity, *ϵ*_*r*_ is the relative permittivity and *μ*_*r*_ is the relative magnetic permeability; the last is assumed to be 1 for all materials throughout. However, when dealing with the insulators, and particularly lossy insulators, it is more convenient and common practice to cast equation (6) in the following form:

B 2 $$\nabla \times (\nabla \times E)-k_{0}^{2}\epsilon_{r}E=0,$$

where *ϵ*_*r*_ is now: $\epsilon_{r}=\epsilon^{\prime }(1-i\;\tan\delta )$. In this expression, *ϵ*^′^ is the real part of the permittivity and tan*δ* is the loss tangent. Now we assume that ***E*** has the form $E(x,y,z)=\tilde{E}(x,y)\;{\text{e}}^{-\text{i}(\beta \hat{z})}$, i.e. that the wire is uniform in the *z*-direction. We can solve equation (7) with the appropriate boundary conditions to obtain the longitudinal complex wavenumber (*β*) of the out-of-plane *z*-direction, which has real part *k* the real wavenumber and imaginary part *α* the longitudinal attenuation.

**(a) Domain and boundary conditions**

The boundary conditions applied when solving the Helmholtz equation in the FEM approach are the following (figure 11). On the outer boundary of the simulation domain surrounding the wires, if *R*, the radius of the circular air region surrounding the wire, is chosen large enough it could be either a perfect electric conductor (PEC; ***n*** × ***E*** = 0) or a perfect magnetic conductor (PMC; ***n*** × ***H*** = 0). However, in practice, *R* is limited by the computational resources and a low reflecting boundary condition or a perfectly matched layer (PML) can be chosen to mimic infinite space. In this work, we chose the latter; in the following, the outermost boundary of the PML is chosen to be PEC, but PMC could equally suffice. It is essential for the existence of the Sommerfeld-type wave that the conductor has a finite resistivity so that the wave has a smaller phase velocity than the corresponding phase velocity in air. For the Goubau wire wave, this is no longer the case, since it was Goubau’s discovery that this condition can be relaxed if the metal wire is coated with a dielectric. Nevertheless, for more realistic results the finite conductivity is important in this case as well. In the GHz range, the skin depth of good conductors, and particularly that of copper, is of the order of 0.1 *μ*m In our case, that is three orders of magnitude smaller than the dimensions of the wire itself. Therefore, the fields effectively only penetrate the wire to this depth. It can be assumed that, in this case, the metal can be replaced with a boundary condition which removes the need to mesh perpendicular to the boundary and resolve the skin depth. In COMSOL, this boundary condition is called the impedance boundary condition (IBC), which allows for finite conductivity and the necessary surface currents to be computed. This condition is used to model the metal wires (copper wire) throughout this work.

The mesh element size is chosen according to the Shannon–Nyquist sampling condition and to resolve the smallest geometry features in the model. In our case, using second-order mesh element shape functions, this translates to a maximum element size of *λ*_0_/6 or *λ*/6, where *λ*_0_ is the free space radiation wavelength and *λ* is the radiation wavelength in a material other than air. Therefore, at 10 GHz the smallest element size with the material properties used is around 5 mm; at 300 GHz it is about 0.1 mm. However, since the wire shape needs to be resolved accurately the smallest size is ultimately set by the geometry constraint. We found that by having 15 mesh elements for each quadrant of the circular cross section of the wire, therefore 60 elements in total around the wire’s perimeter, the resolution is sufficient, as indicated by the mesh convergence test. This results in a smallest linear size of the mesh elements of 0.05 mm. As can be seen in figure 12 (single-wire Sommerfeld case) and in figure 13 (single-wire Goubau case), COMSOL’s meshing algorithm automatically grows the element size from this smallest size to the required size in the uniform air domain surrounding the wire, but this growth rate can be made fully manually controlled. For the PML, because of the underlying coordinate stretching formula, a special mesh type—a mapped mesh—is used, which uses quadraliteral elements. Otherwise, standard triangular elements suffice throughout the model. The colouring scheme in figure 12 indicates the mesh element size of the sides of the triangle (or quadraliteral elements in the PML) measured in millimetres. The mesh is very similar for the Goubau wire case; however, the wavelength in the dielectric is smaller and, consequently, the smallest mesh sizes are 3.8 mm for 10 GHz and 0.08 mm for 300 GHz. However, again, we use 15 elements per quadrant on both sides of the coating (metal and air side), which ultimately sets the smallest linear size of the mesh for Goubau-type wires at 0.05 mm in the frequency range studied in this article; see figure 13.

**(b) Boundary conditions specific for coupled wires**

Following the analytic approach to the coupling presented in the previous section, we then set out to investigate the same configuration numerically with the use of FEM. The equation we are solving in this case is identical to the Helmholtz equation of the single wire (see equation (2)). The boundary conditions for each wire are identical to the ones used in the single-wire wave cases (figure 11); however, now we use the symmetry inherent in the geometry for the two-wire wave case. Only one-half of the geometry is modelled directly and meshed; subsequently equation (2) (Helmholtz equation) is only solved over this half-domain. The full geometry results, for instance field plots, can be recovered in post-processing. This results in a significant reduction in the number of degrees of freedom, thereby reducing the required computational resources and solution time. On the symmetry plane, therefore, we require either a PMC or PEC boundary condition. PEC corresponds to the antisymmetric mode, whereas PMC corresponds to the symmetric mode [25] (figure 14).

## Appendix C. Experimental methods

We used standard scattering parameter (*S*-parameter) measurements. We used a HP/Agilent two-channel, 8722ET Transmission/Reflection Vector Network Analyzer (VNA), with a range of 50 MHz to 40 GHz, with 1601 sampling points in the frequency interval. The schematic of the measurements can be seen in figure 15. The distance between the two SW-carrying wires (indicated SW line1 and SW line2 on the schematic diagram in figure 15) is varied and the *S*-parameter matrix of the system is measured and recorded with the VNA. The coaxial cables connecting the ports of the VNA to the splitters are calibrated out during the calibration process. For the SW mode excitation planar launchers were used, whose design is based on the THz plasmon launchers described by Akalin *et al.* [28]. By applying scaling theory, the dimensions of the launchers are scaled for GHz applications. The SW launchers were designed and manufactured using our in-house facilities. The actual measurement set-up with the SW launchers on the emitter side of the wires is shown in figure 16. The receiver side of the wires is identical to the emitter. The support jig has slots for the launchers on both the emitter and receiver sides, facilitating controlled variation of the separation of the wires. The coaxial cable phase shifters are not shown in figure 16, but were placed according to the schematic drawing shown in figure 15. We separately measured these identical short coaxial cables, and found that their losses can be neglected (0.1 dB each) and that they are identical. By inserting them, we could simultaneously measure the antisymmetric and symmetric two-wire SW depending on the frequency. In order to compare our calculations on the SW coupling, the components of the measurement set-up were measured separately. The *S*-parameters of the splitters and the coaxial cables used to connect them to the launchers were also separately recorded. The *S*-parameters of the launchers were also measured in a back-to-back configuration in which two planar launchers are connected to each other without the wires. These *S*-parameters were added to the calculated two-wire losses and compared with measured *S*_21_ values of the two-wire Goubau waves. The *S*_21_ measurement data of the back-to-back launchers and the splitter circuits are shown in figure 17. The green lines indicate the frequency range in which launchers and splitters perform best in terms of their transmission characteristics. As can be seen in figure 17, the launchers have a peak transmission efficiency (maximum in *S*_21_ or minimum losses) at 4 GHz, at which frequency we expect to have the best match between the calculated and measured results. In addition, in this frequency range, the *S*_21_ parameters of the splitters are very close to a linear function of frequency. These make it possible to take their effect into account by subtracting these values from the whole system’s measured *S*_21_ values (or adding the losses) and see the two-wire wave’s *S*_21_ alone. Indeed it is observed in figure 8 that, in this frequency range, the expected (calculated) values for the *S*_21_ values of the two-wire wave closely match the results from the measurements.
